# A Simple-to-Use Nomogram for Predicting the Survival of Early Hepatocellular Carcinoma Patients

**DOI:** 10.3389/fonc.2019.00584

**Published:** 2019-07-10

**Authors:** Si-Hai Chen, Qin-Si Wan, Di Zhou, Ting Wang, Jia Hu, Yu-Ting He, Hai-Liang Yuan, Yu-Qi Wang, Kun-He Zhang

**Affiliations:** ^1^Department of Gastroenterology, The First Affiliated Hospital of Nanchang University, Jiangxi Institute of Gastroenterology and Hepatology, Nanchang, China; ^2^Department of Occupational and Environmental Health Sciences, School of Public Health, Peking University, Beijing, China

**Keywords:** early hepatocellular carcinoma, nomogram, survival analysis, prediction, clinic utility

## Abstract

**Objective:** This study aimed to develop and validate a simple-to-use nomogram for early hepatocellular carcinoma (HCC) patients undergoing a preoperative consultation and doctors conducting a postoperative evaluation.

**Methods:** A total of 2,225 HCC patients confirmed with stage I or II were selected from the Surveillance, Epidemiology, and End Results database between January 2010 and December 2015. The patients were randomly divided into two groups: a training group (*n* = 1,557) and a validation group (*n* = 668). Univariate and multivariate hazards regression analyses were used to identify independent prognostic factors. The Akaike information criterion (AIC) was used to select variables for the nomogram. The performance of the nomogram was validated concerning its ability of discrimination and calibration and its clinical utility.

**Results:** Age, alpha-fetoprotein (AFP), race, the degree of differentiation, and therapy method were significantly associated with the prognosis of early HCC patients. Based on the AIC results, five variables (age, race, AFP, degree of differentiation, and therapy method) were incorporated into the nomogram. The concordance indexes of the simple nomogram in the training and validation groups were 0.707 (95% CI: 0.683–0.731) and 0.733 (95% CI: 0.699–0.767), respectively. The areas under the receiver operating characteristic (ROC) curve of the nomogram in the training and validation groups were 0.744 and 0.764, respectively, for predicting 3-year survival, and 0.786 and 0.794, respectively, for predicting 5-year survival. Calibration plots showed good consistency between the predictions of the nomogram and the actual observations in both the training and validation groups. Decision curve analysis (DCA) showed that the simple nomogram was clinically useful, and the overall survival significantly differed between low- and high-risk groups divided by the median score of the nomogram in the training group (*P* < 0.001).

**Conclusion:** A simple-to-use nomogram based on a large population-based study is developed and validated, which is a conventional tool for doctors to facilitate the individual consultation of preoperative patients and the postoperative personalized evaluation.

## Introduction

Hepatocellular carcinoma (HCC), accounting for 75–85% of primary hepatic carcinoma (PHC), is the fourth leading cause of cancer death globally in 2018 (1). The prognosis of HCC is associated with the stage of tumor (2). Although early HCC treated with surgical resection has an ~70% of 5-year survival rate (3), the remaining 30% of early HCC patients still have a poor outcome. Therefore, careful evaluation of the prognosis is still needed for early HCC.

Accurate prognostic evaluation is an important step in the management of patients with HCC. The TNM staging system (American Joint Committee on Cancer, AJCC), which was first published in 1977 and is widely used in the clinic, has been updated to the eighth edition (4). However, the TNM staging system did not perform well in HCC prognosis (5). It was found that there was no significant difference in overall survival between stage I and stage II based on the TNM staging system (6), which indicated that this staging system could not predict the personalized prognosis of early HCC. An easy-to-use and personalized scoring system is still needed to predict the prognosis of early HCC.

Recently, the nomogram, a simple and personalized visual tool, has been widely used in the diagnosis and prognosis of diseases (7, 8). Behind the nomogram is a complicated computational formula that can simply determine the definite survival probability. Some scoring systems of the nomogram have demonstrated better performance in predicting prognosis (9–11).

The Surveillance, Epidemiology, and End Results (SEER) program based on the U.S. population provides an abundance of high-quality information for different cancers. Several studies on the prognosis of cancer are based on the SEER database (12, 13). In the present study, information on early HCC was extracted from the SEER database to establish a nomogram that is intuitive and easy to use for predicting the prognosis of early HCC.

## Patients and Methods

### Patients

**Figure 1:**
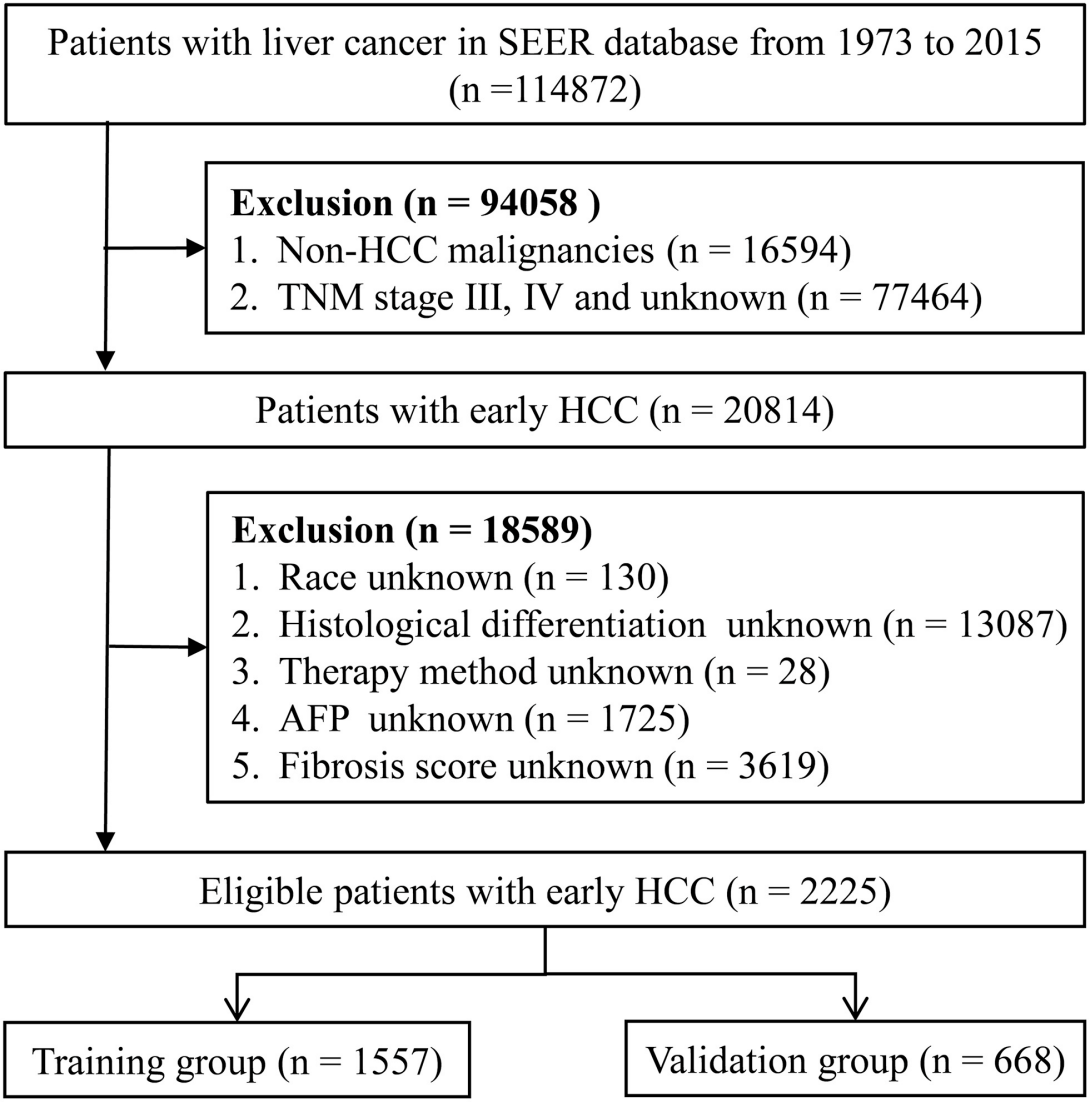
Flow diagram for selecting early HCC patients. SEER, the Surveillance; Epidemiology, and End Results database; HCC, hepatocellular carcinoma; AFP, alpha fetoprotein.

A total of 114,872 patients with liver cancer between 1973 and 2015 were retrospectively extracted from the SEER database. Fifteen variables were selected in this study, including age, sex, race, year of diagnosis, histological type based on the third revision of the International Classification of Diseases for Oncology (ICD-O-3) (14), degree of differentiation, TNM stage (including explicit T, N, and M stage), alpha-fetoprotein (AFP), fibrosis score, therapy method, months of survival, and vital status. Then, early HCC patients from 2010 and 2015 were selected. We selected 20,814 patients with a TNM stage limited to stage I and II who had a confirmed histological type (ICD-O-3 code: 8170, 8171, 8172, 8173, 8174, or 8175). After excluding patients with an unknown race, degree of differentiation, AFP, and fibrosis score, a total of 2,225 early HCC patients were eventually selected for this study. There were 356 patients who received radiofrequency ablation (RFA) out of a total of 397 patients with local tumor treatment. The remaining 41 patients received other treatments including local tumor destruction by ultrasound or acetic acid (14 patients), cryosurgery (7 patients), percutaneous ethanol injection (6 patients), laser (2 patients), electrocautery (1 patient), and unknown local therapy (10 patients) (see Supplementary Table 1 and Supplementary Figure 2). Those 2,225 patients were randomly divided into a training group (*n* = 1557) used to develop the nomogram and a validation group (*n* = 668) used to validate the nomogram. A flow diagram demonstrating the detailed screening process is shown in Figure 1.

### Statistical Analysis

Univariable Cox regression analysis was performed to explore the potential confounders. Subsequently, variables with the *P* value below 0.5 in univariate analysis were selected into multivariable Cox regression to identify the independent predictors (15). The backward step-down process based on the Akaike information criterion (AIC) was used to select the final variables for constructing the nomogram (16, 17). The concordance index (C-index) and receiver operating characteristic (ROC) curve were used to evaluate the discrimination ability of the nomogram. The C-index is defined as the proportion of all evaluable and orderly patient pairs whose predictions are consistent with the results (18). Calibration curves were plotted to assess the calibration ability of the nomogram. Decision curve analysis (DCA) was performed to show the clinical usefulness of the nomogram (19, 20). Kaplan–Meier curves were constructed to analyze the difference in overall survival between the high- and low-risk groups based on the median score of patients in the training group according to the nomogram.

Student's *t*-test and the chi-square test were used to compare the differences between the training and validation groups for the continuous and categorical variables, respectively. SPSS software (IBM Corporation, USA, version 24) was used for the randomization of groups and univariate and multivariate Cox proportional hazards regression analyses. The cutoff value for age was determined by the X-tile program (21). The R statistical packages “rms,” “survival,” “foreign,” and “survivalROC” were used to calculate the C-index and plot the calibration curves, the ROC curves, and Kaplan–Meier curves. The source file “stdca.r” was obtained from the website www.mskcc.org, which was used to draw the DCA curves. Two-sided *P* value < 0.05 was considered statistically significant.

## Results

### Demographic and Clinical Characteristics

The demographics and clinical characteristics of the whole, training, and validation groups are shown in Table 1. In the whole group, nearly 76% of patients were male, and ~67% of patients were white. Most patients had an early AJCC stage (stage I), a high fibrosis score, and a positive AFP. Moderately differentiated tumors accounted for more than half of all cases. Across the entire study population, more than 53% of patients underwent surgery. There was no significant difference between the two groups in demographics and clinical characteristics.

**Table 1:** Demographics and clinical characteristics of eligible patients with early HCC.

	**All patients** **(*n* = 2,225)**	**Training group** **(*n* = 1,557)**	**Validation group** **(*n* = 668)**	***P* value**
Age (mean ± SD), years	62.70 ± 9.97	62.72 ± 9.97	62.66 ± 9.96	0.907^a^
**Sex**, ***n*** **(%)**				
Male	1,676 (75.3)	1,161 (74.6)	515 (77.1)	0.205^b^
Female	549 (24.7)	396 (25.4)	153 (22.9)	
**Race**, ***n*** **(%)**				
Black	283 (12.7)	197 (12.7)	86 (12.9)	0.988^b^
White	1,480 (66.5)	1,036 (66.5)	444 (66.5)	
Other^*^	462 (20.8)	324 (20.8)	138 (20.7)	
**AJCC stage**, ***n*** **(%)**				
I	1,380 (62.0)	965 (62.0)	415 (62.1)	0.948^b^
II	845 (38.0)	592 (38.0)	253 (37.9)	
**N**	All are N0	–	–	–
**M**	All are M0	–	–	–
**AFP**, ***n*** **(%)**				
Negative	855 (38.4)	603 (38.7)	252 (37.7)	0.655^b^
Positive	1,370 (61.6)	954 (61.3)	416 (62.3)	
**Histological differentiation**, ***n*** **(%)**				
I	744 (33.4)	524 (33.7)	220 (32.9)	0.896^b^
II	1,142 (51.3)	799 (51.3)	343 (51.3)	
III and IV	339 (15.2)	234 (15.0)	105 (15.7)	
**Fibrosis score**, ***n*** **(%)**				
0–4	618 (27.8)	432 (27.7)	186 (27.8)	0.962^b^
5–6	1,607 (72.2)	1,125 (72.3)	482 (72.2)	
**Primary tumor therapy**, ***n*** **(%)**				
Surgery	1,197 (53.8)	832 (53.4)	365 (54.6)	
Local therapy	397 (17.8)	287 (18.4)	110 (16.5)	
Non-surgery	631 (28.4)	438 (28.1)	193 (28.9)	

* *Other comprises American Indian/Alaska Native, and Asian/Pacific Islander*.

a *t-test, comparison between the training group and the validation group*.

b *Chi-square test, comparison between the training group and the validation group*.

*AFP, alpha fetoprotein; AJCC, American Joint Committee on Cancer; HCC, hepatocellular carcinoma; SD, standard deviation*.

### Prognostic Factors in Patients With Early HCC

In univariate regression analysis, seven variables, i.e., age, race, AJCC stage, AFP level, histological differentiation, fibrosis score, and therapy method, were significantly associated with the overall survival. In multivariate Cox regression analysis, five variables, i.e., age, race, AFP level, histological differentiation, and therapy method, were identified as independent prognostic factors of early HCC (Table 2).

**Table 2:** Univariate and multivariate Cox regression analyses of prognostic factors in patients with early HCC.

**Variable**	**Univariate analysis**		**Multivariate analysis**	
	**HR (95% CI)**	***P* value**	**HR (95% CI)**	***P* value**
**Age**	1.023 (1.015–1.031)	< 0.001	1.018 (1.010–1.026)	< 0.001
**Race**				
Other^*^	Reference		Reference	
Black	1.584 (1.228–2.044)	< 0.001	1.368 (1.055–1.773)	0.018
White	1.384 (1.139–1.683)	0.001	1.259 (1.033–1.535)	0.022
**Sex**				
Male	Reference		Reference	
Female	0.933 (0.789–1.104)	0.421	0.888 (0.749–1.054)	0.175
**AJCC stage**				
I	Reference		Reference	
II	1.227 (1.064–1.414)	0.005	1.154 (0.998–1.334)	0.054
**AFP**				
Negative	Reference		Reference	
Positive	1.424 (1.223–1.657)	< 0.001	1.270 (1.082–1.491)	0.003
**Histological grade**				
I	Reference		Reference	
II	1.071 (0.910–1.259)	0.410	1.362 (1.152–1.610)	< 0.001
III and IV	1.644 (1.342–2.015)	< 0.001	2.032 (1.647–2.507)	< 0.001
**Fibrosis score**				
0–4	Reference		Reference	
5–6	1.453 (1.225–1.722)	< 0.001	1.068 (0.895–1.276)	0.465
**Primary tumor therapy**				
Resection	Reference		Reference	
Local therapy	2.099 (1.717–2.567)	< 0.001	2.116 (1.722–2.601)	< 0.001
Non-surgery	4.358 (3.706–5.125)	< 0.001	4.304 (3.628–5.106)	< 0.001

* *Other comprises American Indian/Alaska Native, and Asian/Pacific Islander*.

*AFP, alpha fetoprotein; AJCC, American Joint Committee on Cancer; HCC, hepatocellular carcinoma*.

### Construction and Validation of the Prognostic Prediction Nomogram

**Figure 2:**
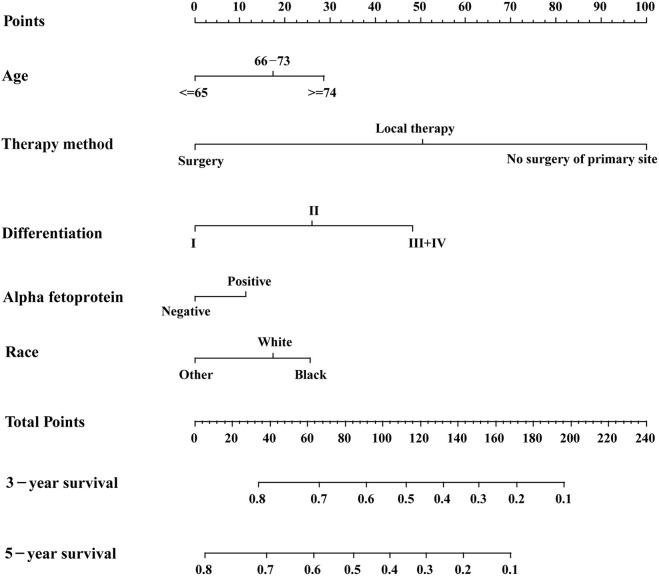
The nomogram for predicting 3- and 5-year survival probabilities of patients with early hepatocellular carcinoma.

Based on the AIC results, race, age, AFP, histological differentiation, and therapy method were identified as variables that were incorporated into the nomogram (Figure 2), which is an intuitive visualization of the model. According to the nomogram, therapy method had the greatest influence on the prognosis of early HCC, followed by histological differentiation, age, race, and AFP. Users could determine the total score based on the individual scores of those five variables and obtain a specific probability of 3- and 5-year survival. The detailed scores of all variables are shown in Table 3.

**Table 3:** Detailed scores of all variables in the nomogram.

**Variable**	**Nomogram score**
**Age (years)**	
≤65	0
66–74	17
≤75	29
**Race**		
Other^*^	0
White	17
Black	25
**AFP**	
Negative	0
Positive	11
**Differentiation**	
I	0
II	26
III and IV	48
**Therapy method**	
Surgery	0
Local therapy	51
No surgery of primary site	100

* *Other comprises American Indian/Alaska Native and Asian/Pacific Islander*.

*AFP, alpha fetoprotein*.

**Figure 3:**
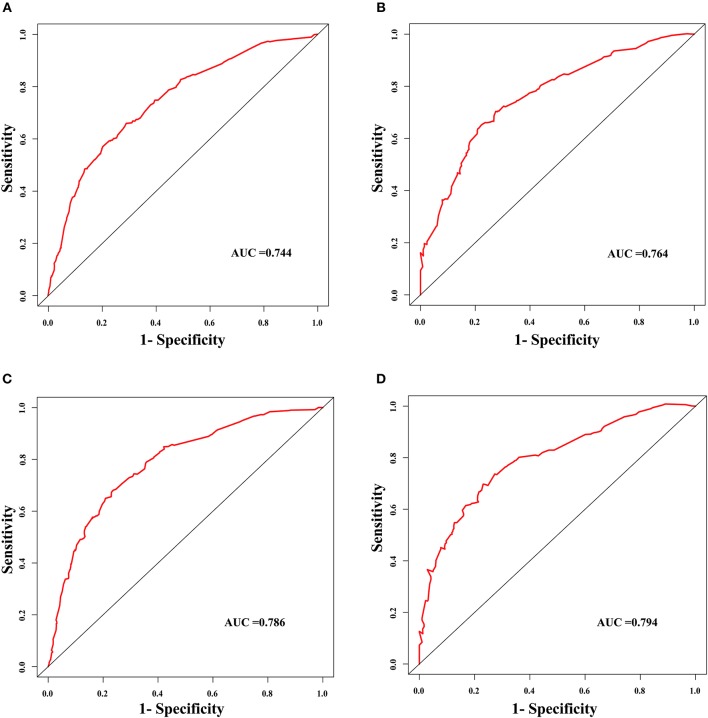
The ROC curves of the nomogram for the prognostic prediction of early hepatocellular carcinoma. **(A)** For predicting 3-year survival in the training group. **(B)** For predicting 3-year survival in the validation group. **(C)** For predicting 5-year survival in the training group. **(D)** For predicting 5-year survival in the validation group. AUC, area under the receiver operating characteristic curve.

**Figure 4:**
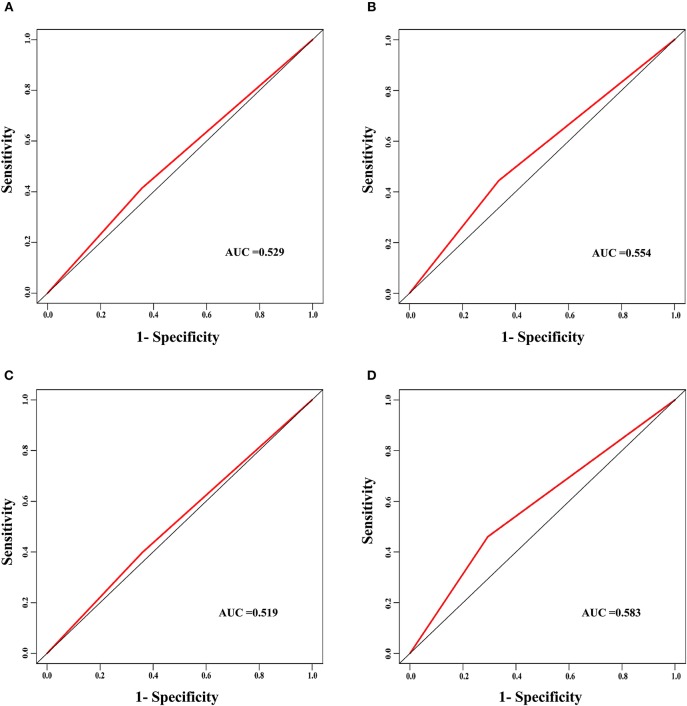
The ROC curves of the TNM stage for the survival prediction of early hepatocellular carcinoma. **(A)** For predicting 3-year survival in the training group. **(B)** For predicting 3-year survival in the validation group. **(C)** For predicting 5-year survival in the training group. **(D)** For predicting 5-year survival in the validation group. AUC, area under the receiver operating characteristic curve.

**Figure 5:**
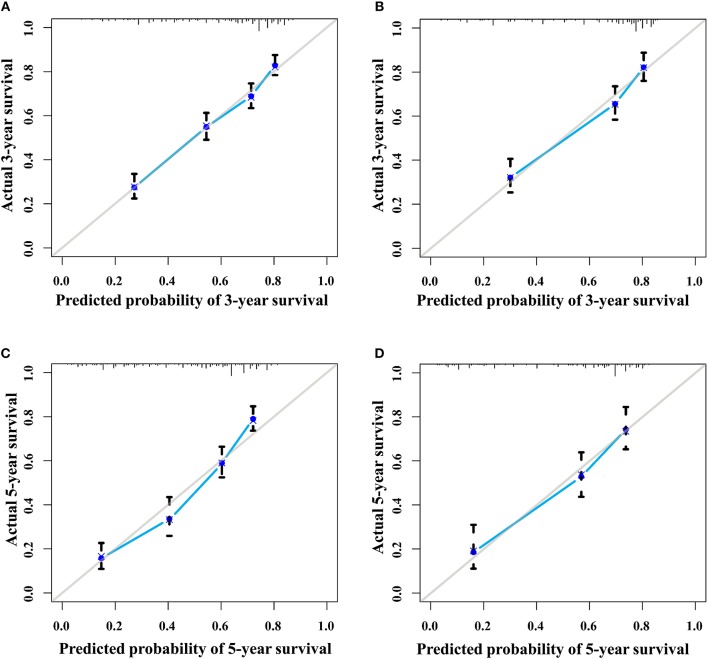
The calibration curves of the nomogram for 3- and 5- year survival probabilities. **(A)** Three-year survival for the training group. **(B)** Three-year survival for the validation group. **(C)** Five-year survival for the training group. **(D)** Five-year survival for the validation group.

The discrimination power of the nomogram was evaluated by the C-index values and ROC curves. The C-indexes for the prediction of overall survival in the training and validation groups were 0.707 (95% CI: 0.683–0.731) and 0.733 (95% CI: 0.699–0.767), respectively. However, the C-indexes of the TNM staging system in the training and validation groups were 0.511 (95% CI: 0.488–0.534) and 0.546 (95% CI: 0.511–0.581). The areas under ROC curve (AUROCs) of the 3-year survival probability in the training and validation groups were 0.744 and 0.764, respectively (Figures 3A,B). The AUROCs of the 5-year survival probability in the training and validation groups were 0.786 and 0.794, respectively (Figures 3C,D). AUROCs of TNM stages were 0.529 and 0.554, respectively, for predicting 3-year survival probability in the training and validation groups (Figures 4A,B), and 0.519 and 0.583, respectively, for predicting 5-year survival probability in the training and validation groups (Figures 4C,D). The calibration curves of the nomogram showed good probability consistencies between the prediction and observation (Figure 5).

### Clinical Value of the Nomogram

**Figure 6:**
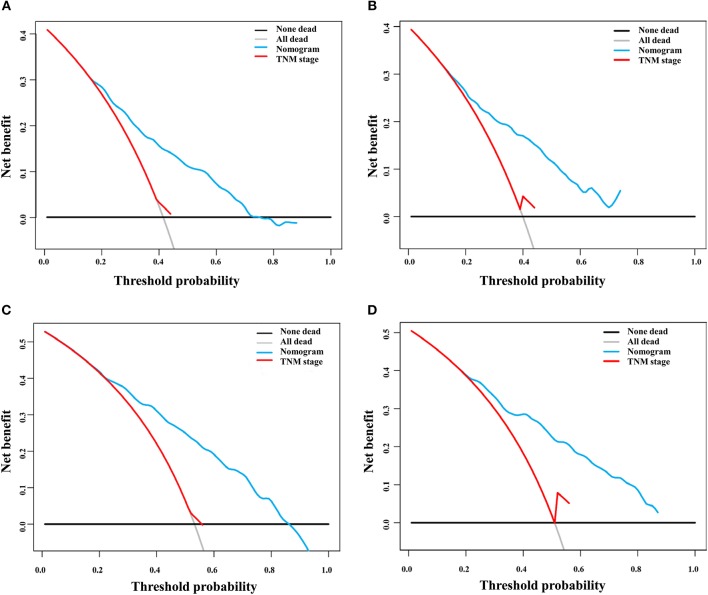
Decision curve analysis of the nomogram and TNM staging system for the survival prediction of patients with early hepatocellular carcinoma. **(A)** Three-year survival in the training group. **(B)** Three-year survival in the validation group. **(C)** Five-year survival in the training group. **(D)** Five-year survival in the validation group.

**Figure 7:**
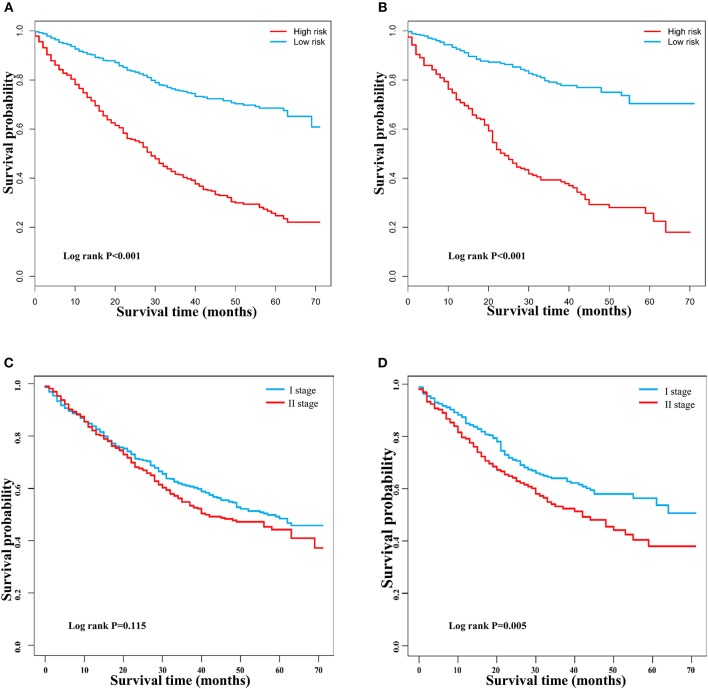
Kaplan–Meier survival curves of patients with early hepatocellular carcinoma stratified by the nomogram and TNM staging. **(A)** Training group based on the nomogram. **(B)** Validation group based on the nomogram. **(C)** Training group based on TNM staging. **(D)** Validation group based on TNM staging.

DCA is a novel method for evaluating alternative prognostic strategies, which has advantages over AUROC (19, 20). The 3- and 5-year DCA curves for the nomogram and the AJCC stage in both the training and validation groups are presented in Figure 6. Compared with the AJCC stage, the nomogram had higher net benefits, which indicated that it had better clinical utility. We divided the early HCC patients into two different risk groups based on the median score of the nomogram, 76 points, in the training group, and the high-risk group had a lower survival probability in both the training and validation groups (Figures 7A,B). There was no difference between patients with TNM stage I and II in the training group, but patients with TNM stage I had higher survival rate than stage II in the validation group (Figures 7C,D).

## Discussion

In this study, we first established a nomogram to evaluate the definite 3- and 5-year survival probabilities of early HCC patients based on a population-based database and verified the ability of the nomogram regarding its discrimination and calibration in both training and validation groups. The performance of the nomogram in the validation group was better than that in the training group, which indicated that the nomogram might have a better external utility. In the perspective of clinical utility, the nomogram had a wide range of threshold probabilities. Additionally, our results also showed that the TNM staging system could not accurately reflect the exact survival probability in early HCC, which was consistent with the findings of other studies (6). Moreover, the nomogram divided the patients with early HCC into two groups, the low- and high-risk groups, which had significant difference in overall survival. These results indicate that the nomogram could be used as a conventional tool in predicting the prognosis of early HCC.

In the present study, age was an independent prognostic factor of early HCC. A patient with younger age showed an increased overall survival. Other studies on the prognosis of early HCC also found that patients with a younger age had a better prognosis, even in the elderly (22, 23).

Race is a controversial prognostic factor. An early study found that there was no significant difference of survival time between black and white patients (24). A study based on SEER database showed that blacks had the highest 1- and 3-year mortality rates, followed by Hispanics, whites, and Asians (25). Our study also found that blacks had a higher mortality rate than whites, while the “other” race, including 425 Asians or Pacific Islanders and 37 American Indians/Alaska Natives, had the lowest mortality. In addition, we found that there was no difference of survival time between 425 Asians or Pacific Islanders and 37 American Indians/Alaska Natives (see Supplementary Figure 1).

The degree of differentiation is a critical characteristic of carcinoma. Nathan et al. (26) selected 788 patients with early HCC and found that tumor histologic grade was not an independent prognostic factor in early HCC, but patients with poor differentiation had a worse survival than patients with a high degree of differentiation. However, this study did not exclude the degree of unknown differentiation. Another study established a survival prediction model for patients with postoperative HCC and identified that the degree of differentiation was an independent predictor for the 5-year survival rate of HCC patients (27). Some studies also found that poor differentiation was associated with HCC recurrence after curative hepatectomy and liver transplantation (28, 29). In our study, the patients with unknown degree of differentiation were excluded, and the results showed that a worse degree of differentiation was associated with a poor prognosis.

The option of surgery or local destruction is controversial in patients with early HCC. A study based on SEER demonstrated that patients with surgical resection had a longer lifetime than those with thermal ablation (30). A propensity-matched analysis in a single institution compared hepatectomy with stereotactic body radiotherapy and concluded that patients who received surgical resection had a higher overall survival (31). Another study that included 7,185 patients with a tumor size ≤ 3 cm showed that surgical resection might provide a lower rate of recurrence than percutaneous ablation, but there were no differences between surgical resection, RFA, and percutaneous ethanol ablation and overall survival (32). Similarly, a retrospective study found that hepatectomy did not provide a higher overall survival compared with RFA combined with chemoembolization in patients with early HCC (33). In particular, the treatment methods that early HCC patients received are influenced by several factors, such as age, income, tumor characteristics, liver-related comorbidities, and hospital factors (34). However, we proposed that if the patient condition allows, surgery is still the preferred treatment.

AFP has been widely used not only for diagnosis but also for predicting the prognosis of HCC. A large tumor size, bilobar involvement, massive or diffuse types, and portal vein thrombosis might contribute to the high levels of AFP (35). In fact, AFP is still a controversial biomarker for HCC (36, 37). Giannini et al. (38) found that AFP was not associated with the prognosis of well-compensated cirrhosis patients with single and small HCC. In this study, we found that AFP was an independent prognostic factor. More clinical studies are needed to estimate the prognostic significance of AFP in early HCC.

Several staging systems have been developed to stratify the patients with HCC, including Barcelona Clinic Liver Cancer (BCLC) staging (39), Cancer of the Liver Italian Program score (40), Japan Integrated Staging Score (41), and Chinese University Prognostic Index (CUPI) (42). These staging systems are frequently utilized for prognostic evaluation for all stages of HCC. Compared with these systems, our nomogram exhibits better predictive value for the overall survival of early HCC. A study including 232 patients with early HCC showed that the C-indexes and AUROCs were 0.6479 and 0.5949 in the training group and 0.6323 and 0.5873 in the validation group, respectively, for BCLC staging, and 0.6096 and 0.5231 in the training group and 0.6889 and 0.5714 in the validation group, respectively, for CLIP staging (43). Nathan et al. (44) found that the C-indexes of CLIP, BCLC, JIS, and AJCC staging systems were 0.51, 0.51, 0.52, and 0.59, respectively, in 379 patients with early HCC.

Wan et al. (45) developed a prognostic nomogram based on 661 stages of HCC patients and validated it by 220 patients in a single institution, in which the total C-indexes of training and validation groups were 0.81 and 0.78, respectively, higher than the current staging systems (TNM, BCLC, Okuda, JIS, CLIP, and CUPI), but the C-index for early stage HCC was not known. Compared with their nomogram, ours had fewer variables and was population-based, including 1557 patients in the training set and 668 patients in the validation set.

Our nomogram only contained five variables, which represented a simple and visual tool for the prognostic prediction of patients with early HCC. To the best of our knowledge, this is the first nomogram for predicting the survival of early HCC patients. Before treatment option, the nomogram could be used to select therapy methods and predict survival rates. After treatment, the nomogram could help doctors to distinguish high- and low-risk patients, and careful follow-up should be performed in high-risk patients.

However, this study still has some limitations. Although the performance of the nomogram was better in the validation group, multicenter clinical application is also needed to evaluate the external utility of this nomogram. Most patients with HCC are associated with chronic liver disease (46), and underlying liver function is possibly an important factor for predicting the prognosis of HCC. Because of the lack of information on liver function in SEER, this nomogram did not contain any variables on liver function, which may be useful to modify the monogram.

## Conclusion

In conclusion, we developed and validated a nomogram for predicting the personalized survival probability of early HCC patients. The simple nomogram had an adequate ability of discrimination and calibration, and good clinical utility. It could be a useful tool for patients undergoing a preoperative consultation and doctors conducting a postoperative evaluation.

## Data Availability

Publicly available datasets were analyzed in this study. This data can be found here: https://seer.cancer.gov/.

## Author Contributions

S-HC and K-HZ contributed to the idea and design. S-HC, Q-SW, DZ, and K-HZ contributed to the data acquisition and analysis. S-HC, Q-SW, TW, and K-HZ contributed to the manuscript writing and revision. All authors contributed to data acquisition and analysis and to manuscript writing and revision, and agreed to all aspects of the work.

### Conflict of Interest Statement

The authors declare that the research was conducted in the absence of any commercial or financial relationships that could be construed as a potential conflict of interest.
