# Supplementary material for: A Simple-to-Use Nomogram for Predicting the Survival of Early Hepatocellular Carcinoma Patients
Source: Front Oncol. 2019 Jul 10;9:584. doi: 10.3389/fonc.2019.00584 (PMC6635555; doi:10.3389/fonc.2019.00584)
Supplement: Supplementary file 1 [file Data_Sheet_1.docx]

**Table 1.** Demographics and clinical characteristics of patients treated with local therapy

|  | All patients  (n=397) | Ablation (n=356) | Other treatments ^a^ (n=41) | P value |
| --- | --- | --- | --- | --- |
| **Age (Mean±SD), years** | 63.66 **±** 9.05 | 63.49 **±** 9.02 | 65.12 **±** 9.24 | 0.276 ^c^ |
| **Sex, n (%)** |  |  |  |  |
| Male | 305 (76.8) | 269 (75.6) | 36 (87.8) | 0.079 ^d^ |
| Female | 92 (23.2) | 87 (24.4) | 5 (12.2) |  |
| **Race, n (%)** |  |  |  |  |
| Black | 46 (11.6) | 40 (11.2) | 6 (14.6) | 0.727 ^d^ |
| White | 274 (69.0) | 244 (68.5) | 30 (73.2) |  |
| Other ^b^ | 77 (19.4) | 72 (20.2) | 5 (12.2) |  |
| **AFP, n (%)** |  |  |  |  |
| Negative | 146 (36.8) | 129 (36.2) | 17 (41.5) | 0.511 ^d^ |
| Positive | 251 (63.2) | 227 (63.8) | 24 (58.5) |  |
| **Histological differentiation, n (%)** |  |  |  |  |
| I | 152 (38.3) | 134 (37.6) | 18 (43.9) | 0.675 ^d^ |
| II | 200 (50.4) | 182 (51.1) | 18 (43.9) |  |
| III and IV | 45 (11.3) | 40 (11.2) | 5 (12.2) |  |
| **Fibrosis score, n (%)** |  |  |  |  |
| 0-4 | 73 (18.4) | 64 (18.0) | 9 (22.0) | 0.534 ^d^ |
| 5-6 | 324 (81.6) | 292 (82.0) | 32 (78.0) |  |

**Note** a : Other treatments comprise local tumor destruction by ultrasound or acetic acid, cryosurgery, percutaneous ethanol injection, laser, electrocautery and unknown local therapy; b : the other race comprises American Indian/Alaska Native, Asian/Pacific Islander; c: t test, comparison between training group and validation group; d: Chi-Squared test, comparison between training group and validation group.

**Abbreviation:** AFP, alpha fetoprotein; SD, standard deviation.


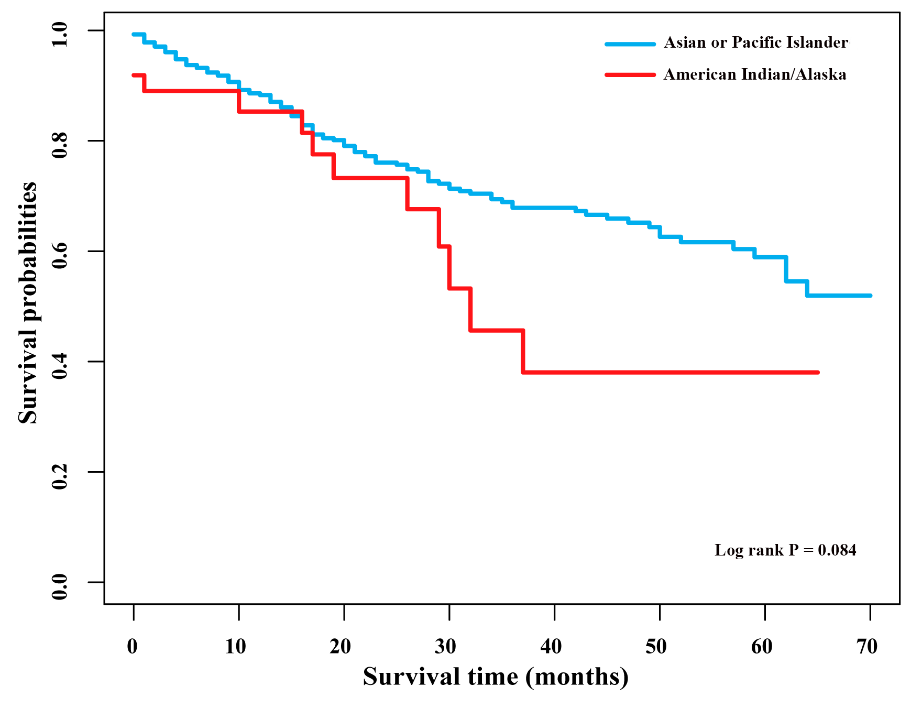


**Figure 1.** Kaplan–Meier curves of Asian or Pacific Islander and American Indian/Alaska.


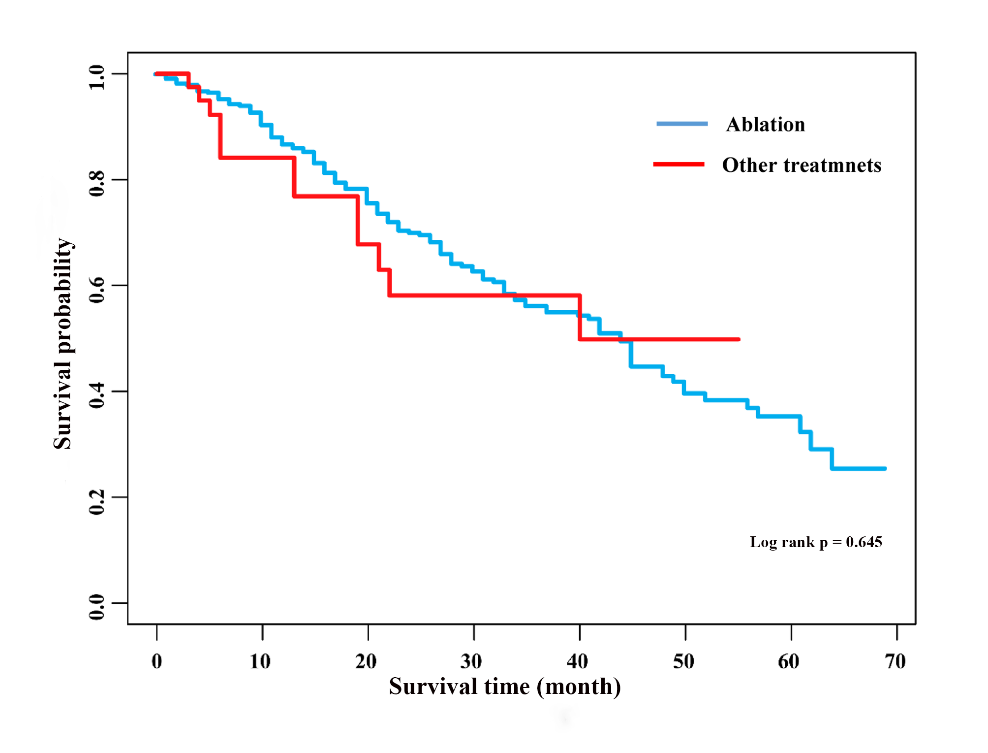


**Figure 2.** Kaplan–Meier curves of ablation and other treatments for HCC.
